# Supplementary material for: Randomised, controlled, open label, multicentre clinical trial to explore safety and efficacy of hyperbaric oxygen for preventing ICU admission, morbidity and mortality in adult patients with COVID-19
Source: BMJ Open. 2021 Jul 5;11(7):e046738. doi: 10.1136/bmjopen-2020-046738 (PMC8260306; doi:10.1136/bmjopen-2020-046738)
Supplement: Supplementary data [file bmjopen-2020-046738supp001.pdf]

Study Code:	COVID-19-HBO
Version No:	v.4
Date:	2021-02-27
EudraCT No:	2020-001349-37

CLINICAL STUDY PROTOCOL

A Randomized, Controlled, Open Label, Multicentre Clinical Trial to explore Safety and Efficacy of Hyperbaric Oxygen for preventing ICU admission, Morbidity and Mortality in Adult Patients With COVID-19

Safety and Efficacy of Hyperbaric oxygen for ARDS in patients with COVID-19

Study code:	COVID-19-HBO
EudraCT number:	2020-001349-37
ClinicalTrials.gov Identifier:	NCT04327505
Version number:	4
Date:	2021-02-27
Sponsor:	Karolinska Institutet, Solna
Coordinating Investigator	Anders Kjellberg, MD

Kjellberg A, *et al. BMJ Open* 2021; 11:e046738. doi: 10.1136/bmjopen-2020-046738

Study Code: COVID-19-HBO
Version No: v.4
Date: 2021-02-27
EudraCT No: 2020-001349-37

Table of Contents

Signature page 5
Contact information 7
List of used acronyms and abbreviations 9
1. Synopsis 11
2. Background and Rationale 14
2.1 Clinical manifestations and challenges with COVID-19 14
2.2 Rationale for the study and explanation of the hypothesis 15
3. Benefit-risk evaluation 16
3.1 The risk group 16
3.2 General risks with HBO and oxygen toxicity 17
3.3 Blood sampling 17
3.4 Handling of sensitive personal data 17
3.5 Safety and logistics 18
4. Study objectives 18
4.1 Primary objective 19
4.2 Secondary objective(s) 19
4.2.1 Main secondary objective 19
4.2.2 Other secondary objectives 19
4.3 Primary endpoint: 19
4.4 Secondary endpoints: 20
4.4.1.2 Other Efficacy Endpoints 20
5. Study design and procedures 21
5.1 Overall Study design 21
5.2 Procedures and flow chart 22
5.2.1 Study schedule 22
5.2.2 Assessments and procedures 24
5.3 Biological sampling procedures 30
5.3.1 Handling, storage, and destruction of biological samples 30
5.3.2 Total volume of blood per subject 30
5.3.3 Biobank 30
5.3 End of Study 31
6. Subject selection 31

6.1 Inclusion criteria: 31
6.2 Exclusion criteria: 32
6.3 Screening 32
6.4 Withdrawal Criteria 32
7. Study treatments 33
7.1 Description of investigational product(s) 33
7.2 Dose and administration 34
7.3 Packaging, labeling, and handling of investigational products(s) 34
7.4 Drug accountability and treatment compliance 34
7.5 Randomization 34
7.6 Concomitant Medication 35
7.7 Treatment after study end 35
8. Handling of Adverse Events 35
8.1 Definitions 35
8.1.1 Adverse Event (AE) 35
8.1.2 Adverse Reaction (AR) 35
8.1.3 Serious Adverse Event (SAE) 35
8.1.4 Suspected Unexpected Serious Adverse Reaction (SUSAR) 36
8.2 Assessment of Adverse Events 36
8.2.1 Assessment of causal relationship 36
8.2.2 Assessment of intensity 37
8.2.3 Assessment of seriousness 37
8.3 Reporting and registration of Adverse Events 37
8.3.1 Reporting of Adverse Events (AE) 38
8.3.2 Reporting of Serious Adverse Events (SAE) 38
8.3.3 Reporting of Suspected Unexpected Serious Adverse Reactions (SUSAR) 38
8.4 Follow-up of Adverse Events 38
8.5 Safety Report (Development Safety Update Report, DSUR) 39
8.6 Procedures in case of emergencies 39
8.7 Reference Safety Information 39
9. Statistics 39
9.1 Statistical Analysis Plan 39
9.1.1 Analysis population 40

9.2 Statistical analyses 40
9.2.1 Sample size calculations 40
9.2.2 General statistical methodology 40
9.2.3 Patient Demographic and Baseline Characteristics 41
9.2.4 Primary Endpoint Analysis 41
9.2.5 Secondary Endpoints Analysis 41
9.2.6 Safety analyses 41
9.2.7 Interim Analysis 42
10. Quality Control and Quality Assurance 42
10.1 Quality Assurance and Sponsor oversight 42
10.2 Monitoring 43
10.3 Source data 43
10.4 Deviations or serious breaches 43
10.5 Audits and inspections 43
10.6 Data Safety Monitoring Board 44
10.7 Data protection 44
11. Ethics 45
11.1 Compliance to the protocol, GCP and regulations 45
11.2 Ethical review of the study 45
11.3 Procedure for obtaining informed consent 45
12. Insurances 46
13. Substantial changes to the study 46
14. Collection, handling and archiving data 46
14.1 Case Report Form 46
15. Notification of study completion, reporting, and publication 47
16. References 47
17. Amendments and Administrative changes 50

Study Code: COVID-19-HBO
Version No: v.4
Date: 2021-02-27
EudraCT No: 2020-001349-37

Signature page

Sponsor

I am responsible for ensuring that this protocol includes all essential information to be able to conduct this study. I will submit the protocol and all other important study-related information to the responsible investigator(s) so that they can conduct the study correctly. I am aware that it is my responsibility to hold the staff members who work with this study informed and trained.

2021-03-01

Sponsor's representative signature Date

Peter Lindholm, MD, PhD

Printed name

Coordinating Investigator

I have read this protocol and agree that it includes all essential information to be able to conduct the study. By signing my name below, I agree to conduct the study in compliance with this protocol, the Declaration of Helsinki, ICH GCP (Good Clinical Practice) guidelines and the current national and international regulations governing the conduct of this clinical trial.

I will submit this protocol and all other important study-related information to the staff members and investigators who participate in this study, so that they can conduct the study correctly. I am aware of my responsibility to continuously keep the staff members and investigators who work with this study informed and trained.

I am aware that quality control of this study will be performed in the form of monitoring, audit, and possibly inspection.

2021-03-01

Coordinating Investigator's signature Date

Anders Kjellberg, MD

Printed name

Kjellberg A, *et al. BMJ Open* 2021; 11:e046738. doi: 10.1136/bmjopen-2020-046738

Principal Investigator

I have read this protocol and agree that it includes all essential information to be able to conduct the study. By signing my name below, I agree to conduct the study in compliance with this protocol, the Declaration of Helsinki, ICH GCP (Good Clinical Practice) guidelines and the current national and international regulations governing the conduct of this clinical trial.

I will submit this protocol and all other important study-related information to the staff members and investigators who participate in this study, so that they can conduct the study correctly. I am aware of my responsibility to continuously keep the staff members and investigators who work with this study informed and trained.

I am aware that quality control of this study will be performed in the form of monitoring, audit, and possibly inspection.

[signature] 2021-03-01

Principal Investigator's signature Date

ANDERS KJELLBERG

Printed name

Study Code: COVID-19-HBO
Version No: v.4
Date: 2021-02-27
EudraCT No: 2020-001349-37

Contact information

**Role**	
Sponsor	Karolinska Institutet<br><br>Sponsor representative:<br>Peter Lindholm, MD, PhD<br>Hyperbaric Medicine,<br>Dept. Physiology and Pharmacology<br>Karolinska Institutet<br>171 77 Stockholm<br>+46730621184<br>peter.lindholm@ki.se
Coordinating Investigator Karolinska Institutet/ Principal Investigator Karolinska University Hospital	Anders Kjellberg, MD, PhD student,<br>ICU Consultant, head of hyperbaric unit,<br>Perioperative Medicine och Intensive Care<br>Karolinska University Hospital/<br>Hyperbaric Medicine<br>Dept. Physiology and Pharmacology<br>Karolinska Institutet<br>171 77 Stockholm<br>+468760657355<br>anders.kjellberg@ki.se
Principal investigator<br>Blekingesjukhuset, Karlskrona	Johan Douglas, MD<br>Anestesikliniken<br>Blekingesjukhuset<br>Karlskrona<br>+46708123223<br>j@douglas.nu
National Coordinating Investigator Germany/ Principal Investigator<br>St. Josef, Regensburg	Michael Pawlik, MD, PhD<br>Klinik für Anästhesiologie, Krankenhaus St.Josef<br>93053 Regensburg<br>+499417823610<br>mpawlik@caritasstjosef.de
Principal Investigator Sahlgrenska University Hospital	Louise Sameby, MD<br>Department of Anesthesia and Intensive Care,<br>Hyperbaric Medicine<br>louise.sameby@vgregion.se
Trial sites	Blekingesjukhuset Karlskrona, SE<br>Karolinska University Hospital, SE<br>Sahlgrenska University Hospital, SE<br>Krankenhaus St.Josef, Regensburg, DE

**Role**	
	Bergmannsheil und Kinderklinik Buer, DE
Clinical monitoring organization, Sweden	Karolinska Trial Alliance, KTA Support<br>Karolinska University Hospital Sabbatsbergs sjukhus<br>Olivecronas väg 15<br>113 61 Stockholm, Sweden
Senior Biostatistician	Jan Kowalski<br>JK Biostatistics AB<br>Karlbergsvägen 74<br>113 35 Stockholm, Sweden
Data Safety Monitoring Board	Magnus Nord, MD, PhD, Professor, Astra Zeneca<br>Miklos Lipcsey, MD, PhD, Professor, Uppsala University Hospital<br>Anders Öwall, MD, PhD, Ass. Professor/ Senior Consultant Karolinska Institutet/ Karolinska University Hospital

List of used acronyms and abbreviations

Abbreviation	Term/Explanation
ABG	Arterial Blood Gas
AE	Adverse Event = any untoward medical occurrence
ALI	Acute Lung Injury
ANCOVA	Analysis of Covariance
AR	Adverse Reaction = adverse event, that is each unfavorable and unexpected reaction to a study treatment, regardless of dose
ARDS	Acute Respiratory Distress Syndrome
ATA	Atmosphere Absolute (pressure) 1ATA=101.3kPa
CBG	Capillary Blood Gas
COPD	Chronic Obstructive Pulmonary Disease
COVID-19	Corona Virus Disease 2019
CPTD	Cumulative Pulmonary Toxicity Dose
CRA	Clinical Research Associate
CRF	Case Report Form
CRO	Contract Research Organization
CRRT	Continuous Renal Replacement Therapy
CT	Computerized Tomography
CXR	Chest X-Ray
DNR	Do Not Resuscitate
DSMB	Data Safety Monitoring Board
DSUR	Development Safety Update Report = annual safety report
ECG	Electrocardiogram
ECMO	Extra-Corporal Membrane Oxygenation
EPM	Etikprövningsmyndigheten (English: Swedish Ethical Review Authority)
FAS	Full Analys Set
GCP	Good Clinical Practice
ICF	Informed Consent Form
ICH	International Council for Harmonization
ICU	Intensive Care Unit
IEC	Independent Ethics Comittee
IHD	Intermittent Hemo-Dialysis
IL-	Interleukin-
IRB	Institutional Review Board
ITT	Intention-to-treat = including all data from all subjects who have participated in the study
HBO	Hyperbaric Oxygen
HIF	Hypoxia Inducible Factor
LUS	Lung Ultrasound

Kjellberg A, et al. BMJ Open 2021; 11:e046738. doi: 10.1136/bmjopen-2020-046738

LVFS	Läkemedelsverkets författningssamling (English: Swedish Medical Products Agency's statutes)
M1	Macrophage phenotype 1; inflammatory
M2	Macrophage phenotype 2; anti-inflammatory
miR-210	MicroRNA 210
miR-34a	MicroRNA 34a
MPA	Medical Products Agency
NEWS	National Early Warning Score
PBMC	Peripheral Blood Mononuclear Cells
PE	Pulmonary Embolism
$PACO_2$	Partial pressure of carbon dioxide in alveoli
PAH_2O	Partial pressure of water vapor in alveoli
PAO_2	Partial pressure of oxygen in alveoli
PaO_2/FiO_2	Partial pressure of oxygen in arterial blood/Fraction of inspired oxygen
PFI	PaO_2/FiO_2 = partial pressure of oxygen in arterial blood/Fraction of inspired oxygen
PP	Per Protocol analysis = including only data from subjects who have completed the study completely in accordance with the protocol, with no deviations from the protocol
PPS	Per Protocol Set
RNA	Ribonucleic acid
SAE	Serious Adverse Event = serious untoward medical occurrence
SAP	Statistical Analysis Plan
SPC or SmPC	Summary of Product Characteristics
SUSAR	Suspected Unexpected Serious Adverse Reaction
SARS-CoV-2	Severe acute respiratory syndrome coronavirus 2
SOP	Standard Operation Procedure
SpO_2	peripheral Oxygen Saturation
TNFα	Tumor Necrosis Factor alpha
UPTD	Units of oxygen Pulmonary Toxicity Dose

Study Code:	COVID-19-HBO
Version No:	v.4
Date:	2021-02-27
EudraCT No:	2020-001349-37

1. Synopsis

EudraCT number:	2020-001349-37
Title:	A Randomized, Controlled, Open Label, Multicentre Clinical Trial to explore Safety and Efficacy of Hyperbaric Oxygen for preventing ICU admission, Morbidity and Mortality in Adult Patients With COVID-19
Study code:	COVID-19-HBO
ClinicalTrials.gov identifier:	NCT04327505
Short background/ Rationale/Aim:	**COVID-19** may cause **severe pneumonitis** that requires ventilatory support in some patients where the **ICU mortality** is as high as 62%. Hospitals do **not** have **enough ICU beds** to handle the demand and to date there is **no effective cure**.<br>We explore a treatment administered in a randomized clinical trial that could **prevent ICU admission** and **reduce mortality**.<br>The overall hypothesis to be evaluated is that HBO reduces mortality, increases hypoxia tolerance and prevents organ failure in patients with COVID19 pneumonitis by attenuating the inflammatory response.
Study objectives:	**Primary objective:**<br>To evaluate if HBO reduces the number of ICU admissions compared to best practice for COVID-19<br>**Main secondary objectives:**<br>To evaluate if HBO reduces the load on ICU resources, morbidity and mortality in severe cases of COVID-19<br>To evaluate if HBO mitigates the inflammatory reaction in COVID-19<br>**Other secondary objectives (in selection):**<br>To evaluate if HBO is safe for SARS-CoV-2 positive patients and staff
Study design:	Randomized, controlled, phase II, open label, multicentre
Study population:	Adult patients with SARS-CoV-2 infection, with at least two risk factor for increased mortality, likely to develop ARDS criteria and need intubation within 7 days of admission to hospital.
Number of subjects:	200 (20+180)
Inclusion criteria:	1) Aged 18-90 years<br>2) PaO_2/FiO_2 (PFI) below 200 mmHg (26.7 kPa) (based on ABG measurement)<br>3) Suspected or verified SARS-CoV-2 infection<br>4) At least two risk factors for increased morbidity/mortality<br>• Age above 50 years<br>• Hypertension

Kjellberg A, *et al. BMJ Open* 2021; 11:e046738. doi: 10.1136/bmjopen-2020-046738

	• Cardiovascular disease<br>• Diabetes or pre-diabetes<br>• Active or cured cancer<br>• Asthma/COPD<br>• Smoking<br>• D-Dimer > 1.0<br>• Auto-immune disease<br>5) Documented informed consent according to ICH-GCP and national regulations
Exclusion criteria:	1) ARDS/pneumonia caused by other viral infections (positive for other virus)<br>2) ARDS/pneumonia caused by other non-viral infections or trauma<br>3) Known pregnancy or positive pregnancy test in women of childbearing age<br>4) Patients with previous lung fibrosis more than 10% (verified by CT)<br>5) CT- or spirometry-verified severe COPD with emphysema<br>6) Contraindication for HBO according to local guidelines<br>7) Not likely to need ICU admission within 7 days of screening (Subjective criteria that may exclude any patients that fullfill the other inclusion criteria but where the treating physician suspect a spontaneous recovery)<br>8) Mental inability, reluctance or language difficulties that result in difficulty understanding the meaning of study participation<br>9) Prisoner (Exclusion criteria according to IRB at UCSD)<br>10) Unable/risky to move patient to Hyperbaric chamber
Investigational product(s), dosage, administration:	Hyperbaric oxygen (HBO) compared with best practice treatment<br>HBO: HBO 1.6-2.4 ATA for 30-60 min, maximum 5 treatments first 7 days<br>Control: Best practice treatment for COVID-19
Study endpoints:	**Primary endpoint:**<br>The proportion of subjects admitted to ICU from day 1 to day 30, based on at least one of the following criteria:<br>i) Rapid progression over hours<br>ii) Lack of improvement on high flow oxygen >40L/min or non invasive ventilation with fraction of inspired oxygen (FiO_2) > 0.6<br>iii) Evolving Hypercapnia or increased work of breathing not responding to increased oxygen despite maximum standard of care available outside ICU<br>iv) Hemodynamic instability or multi organ failure with maximum standard of care available outside ICU<br><br>**Secondary endpoints:**

	Main Secondary Efficacy Endpoints<br><br>I. Proportion of subjects with 30-day mortality, all-cause mortality, from day 1 to day 30.<br>II. Time-to-Intubation, i.e. cumulative days free of invasive mechanical ventilation, from day 1 to day 30<br>III. Time-to-ICU, i.e. cumulative ICU-free days, derived as the number of days from day 1 to ICU, where all ICU-free subjects are censored at day 30.<br>IV. Mean change in inflammatory response from day 1 to day 30.<br>a. White cell count + differentiation<br>b. Procalcitonin<br>c. C-Reactive protein<br>d. Cytokines (IL-6) (if available at local laboratory)<br>e. Ferritin<br>f. D-Dimer<br>g. LDH<br>V. Overall Survival<br><br>Safety Endpoints<br><br>I. The number of subjects, proportion of subjects and number of events of AE.<br>II. The number of subjects, proportion of subjects and number of events of SAE<br>III. The number of subjects, proportion of subjects and number of events of SADR.<br>IV. Mean change in PaO_2/FiO_2 before and after HBO compared to mean variance in PaO_2/FiO_2 in control group during day 1 to day 7.<br>V. Mean change in NEWS before and after HBO compared to mean change in daily NEWS in control group during day 1-day 7.<br>VI. Number of negative events in staff associated with treatment of subject, (e.g. contact with aerosol from subject), number of events from day 1 to day 30 or last day in hospital if subject is discharged earlier, or at withdrawal.
Study period:	Q2 2020 – Q4 2021
Statistical analyses	Primary and secondary endpoints will be evaluated using the ITT population (i.e. all randomized subjects) and the primary endpoint also using the PP population (i.e. all randomized subjects with no major protocol violations). All randomized subjects will be included in the safety population. The primary analysis of the primary endpoint will be performed using the Cochran Mantel Haenszel test adjusting for randomisation strata site and gender.

2. Background and Rationale

2.1 Clinical manifestations and challenges with COVID-19

SARS-CoV-2 was first identified in China in December 2019 and is now identified as the third Corona virus outbreak in 20 years after SARS-CoV in 2003 and MERS in 2012(Yang et al., 2020b). The clinical infectious disease COVID-19 was declared a pandemic by WHO on March 11, 2020, and more than 400 articles have been published and no specific treatment has been successful despite more than 160 clinical trials being registered in March 2020(Arabi et al., 2020). A synchronized immune response is vital in the control and resolution of viral infections. COVID-19 enters human cells through Angiotensin Converting Enzyme 2 (ACE2), abundant in lungs, arteries, heart, kidney and intestines, causing a downstream activation of an inflammatory cascade that activates the innate immune system. In some patients, this activation and resolution is dysregulated, causing a disproportionate reaction, popularly known as cytokine storm(Guo et al., 2020). Antiviral drugs Lopinavir-Ritonavir did not show any significant benefit compared to standard care in a randomized controlled study of 199 patients (Cao et al., 2020).

Clinical experience from China and Italy is already published and even though the overall mortality is low (3.4%) the numbers from critical care are fearsome(Chen et al., 2020, Yang et al., 2020a, Arabi et al., 2020, Grasselli et al., 2020). Mortality rates have been reported as high as 90% in patients developing ARDS in early reports from Wuhan province and more recent reports have reported overall 28-d mortality rates of 61,5% in ICU patients with acute respiratory illness (Yang et al., 2020a) In a recent retrospective cohort study form Wuhan 19% of patients needed mechanical ventilation or ECMO of whom 97% died, SIC! 26% was admitted to the ICU and hospital mortality rate was 28% (Zhou et al., 2020). Mortality rates in ARDS in general are until now decreasing but still very high. A recent systemic overview reported mortality rates since 2010: Overall rates of in-hospital- 45%, ICU- 38% and 28/30-d- 30% (Maca et al., 2017).

ALI associated with COVID-19 differs from other described ARDS with rapidly progressing respiratory failure and fibrosis; post mortem biopsy of pulmonary tissue form a 72 yo man that died three weeks after the onset of symptoms was described as “diffuse alveolar damage, with reactive type II pneumocyte hyperplasia, intra-alveolar fibrinous exudates were present and loose interstitial fibrosis and chronic inflammatory infiltrates” (Zhang et al., 2020). Even patients with mild symptoms who recover from COVID-19 may have significant changes on pulmonary CT-scan, with diffuse ground glass opacities, crazy-paving pattern and consolidation suggesting severe inflammatory involvement (Pan et al., 2020).

2.2 Rationale for the study and explanation of the hypothesis

Macrophages, part of the innate immune system, have become major therapeutic targets in ALI/ARDS. Macrophage activation is involved in the early phase of ARDS (Sulkowski et al., 1997). Alveolar macrophages (AM) are the gate keepers of the innate immune system in the lungs. Upon activation, they secrete several inflammatory cytokines and chemokines including IL-1β, IL-6 and TNF-α, to attract Th1/Th17-cells, new macrophages and neutrophils. AM are also responsible for clearing apoptotic neutrophils when the infection resolves. Proteomics involved in the switch from inflammatory macrophage (M1) to resolving or anti-inflammatory macrophage sub type (M2) was recently described in a human study of ALI/ARDS (Dong et al., 2013). Hypoxia Inducible factors (HIF-1 and HIF-2) and inflammatory factors such as STAT3 and NFκB are important transcription factors involved in macrophage polarization. How and if we can intervene with this intricate network of redox signalling is not clear (Brune et al., 2013).

Hyperbaric oxygen (HBO) has been used for almost a century, initially for decompression sickness (DCS) but it was soon noted that it had several anti-inflammatory effects (Gill and Bell, 2004, Thom, 2011). Recent evidence from animal studies suggests that HBO ameliorates inflammation in DCS-induced ALI through polarization of macrophages from M1 to M2 (Han et al., 2017, Geng et al., 2015). Hyperbaric oxygen has been shown to polarize macrophages from M1 to M2 associated with IL-10 and thereby reducing inflammation (Buras et al., 2006, Oyaizu et al., 2018) and 30-min HBO ex vivo inhibits monocyte IL-1β and TNF-α (Benson et al., 2003).

Patients presenting to the hospital with COVID-19 normally have almost a week of mild or moderate flu-like symptoms but on admission often have an isolated hypoxic respiratory failure. Many patients, despite severe hypoxemia do not have dyspnoea or carbon dioxide retention suggesting a diffuse but moderate alveolar edema and a hypoxic adaptation. Hypoxia is relative to the upregulation of adaptive mechanisms. When medical oxygen is administered for a prolonged period, the adaptive mechanisms are put out of play and might aggravate oxidative stress. Hyperbaric oxygen will give patients a short burst of oxidative stress and re-activate adaptive responses. In a study with healthy volunteers, we have seen that 28-min of HBO changes microRNA-210 (miR-210) and micro-RNA 34a (miR-34a) in peripheral blood mononuclear cells (PBMC) (own unpublished preliminary data). MiR-210 and miR-34a have been shown to micromanage HIF-1 in the regulation macrophage polarization (Weng et al., 2019, Karshovska et al., 2020). Our hypothesis was recently published in a peer-reviewded journal(Kjellberg et al., 2020) and a mini-review article supporting our hypothesis has also been published(Paganini et al., 2020).

Published and unpublished case reports from China and USA indicate that HBO in these patients may be safe and beneficial(Zhong, 2020, Chen, 2020, Thibodeaux et al., 2020). HBO has the potential to reduce inflammation, restore normal defence mechanisms and thereby

reduce morbidity and mortality in COVID-19 pneumonitis. A recent prospective cohort trial showed 50% lower mortality and 65% lower need of mechanical ventilation in the HBO-treated group compared to propensity matched controls(Gorenstein et al., 2020).

3. Benefit-risk evaluation

3.1 The risk group

There is currently no effective treatment available for COVID-19 and the mortality is high in risk groups. The availability of ICU beds with ventilators and other means of supportive care are prognosticated to be exhausted in most countries including Sweden. A recently published case series of five patients “with impeding intubation” supports a previously submitted manuscript and our hypothesis of beneficial effect of HBO for COVID-19, In this case series, all patients recovered with 1-6 treatments, without the need of intubation.

Five trials apart from ours are registred on clinicaltrials.gov. We have communicated with all the principal investigators of the registred trials and several peers that have treated COVID-19 patinents with “compassionate use” of HBO. So far more that 20 patients have been treated with HBO within registred clinical trials and more than 200 patients have been treated on “compassionate grounds” outside clinical trials. Only two incidents of avderse events have been reported, both being desaturation after treatment; one patient required transient non-invasive ventilation and the other one required intubation and mechanical ventilation shortly after HBO. From the “expert opinion” and clinical experience, there are no signals that HBO is overtly dangerous for patients with COVID-19. The only way to scientifically evaluate the safety and efficacy of HBO for COVID-19 is thorugh a well-designed and sufficiently powered clinical trial like ours.

HBO has the potential to prevent COVID-19 infection from developing into ARDS and multiple organ failure which would then relieve ICU resources and potentially save lives. The nature of the disease with high mortality and no effective cure makes the risk group a “vulnerable group” and it is important to make sure that the subjects are not unduly influenced by the expectation or benefits associated with participation. Therefore, we will conduct a clinical trial in compliance with GCP, the Declaration of Helsinki and national regulatory requirements. The written information has a neutral language explaining both risks and potential benefits and investigators are instructed to keep a neutral tone in the oral information.

The cause of the rapid ARDS progression in COVID-19 is still an enigma and the mechanisms of ARDS in general are not fully understood. We present a plausible hypothesis of the mechanism and a possible cure. Since we do not have any better options than to “wait and see”, the potential benefits for the subject outweigh the risk.

3.2 General risks with HBO and oxygen toxicity

There is always a risk of deterioration associated with HBO in these fragile subjects due to the nature of their illness. Hyperbaric oxygen is a well-established method used for almost a century for several different indications. The mechanisms of HBO are not fully understood but it is generally regarded as safe with few adverse events and extremely rare serious adverse events. Undersea and hyperbaric Medical Society (UHMS) has reported a total of 40 complications per 10,000 treatments during 463,293 treatments over the past two years (Moon, 2019). Following are the adverse events per 10,000 treatments: ear pain 20, confinement anxiety 8, hypoglycaemic event 5, shortness of breath 2, seizure 2, sinus pain, 1, chest pain. The rationale for a short treatment in this trial is that there is evidence for effect in 30 minutes and a longer treatment may add to oxygen toxicity. One can argue that the area under the curve is important for effect and hence local variances in dose would result in similar oxygen toxicity, e.g. 1.6-2.0 ATA for 90 minutes would give 144-180 UPTD and 2.4 ATA for 30-60 minutes would give 72-144 UPTD. This needs to be put in relation to the daily dose that these patients receive in normobaric oxygen 40-100%, which is equivalent of 576-1440 CPTU/ 24 hours.

3.3 Blood sampling

Blood sampling may have negative impact on the subject. The subjects are critically ill and would have a large amount of blood sampling daily. Many of the blood sample required for the study are included in the clinical practice, so the actual extra blood taken will in many cases only be half of the volume presented in the procedures. The blood sampling serves three purposes:

1. Safety, which is of benefit for the subject.

2. Efficacy, which at least in part is beneficial for the subject since the exact dose will likely be individual and need to be titrated to effect. It will also serve as a quality control measure to ensure the validity of the data upon presentation of results.

3. Explanatory, which will not benefit the subjects in the present illness but since it is essential to learn more about the COVID-19 disease and HBO, this will potentially benefit the subjects the next time they catch a similar infection. Explanatory objectives are important for public health.

3.4 Handling of sensitive personal data

We will handle personal data including gene expression analyses on the subjects creating a risk of personal integrity violation. The trial is performed according to ICH-GCP, all sites will be informed and educated about the protocol and data will be entered into an eCRF. The data will not identify any person taking part in the study, in accordance with the EU Data Protection Directive (95/46/EU). We have an external monitor that will help us assess the risks by assessing quality of trial design, data collection and informed consent.

3.5 Safety and logistics

There are several safety and logistic issues involved with HBO treatment of subjects with COVID-19 pneumonitis with or without ARDS. Most of the issues are the same as any other patient group, that staff working with HBO is aware of. Subjects will be transported from the ward to the multiplace or monoplace chamber depending on severity on inclusion (according to local guidelines). There are few specific risks with SARS-CoV-2 positive patients that need to be addressed.

1. The risk of viral spread and contamination:
   a. during transport must be addressed according to local guidelines to minimize contact with personell and other patients.
   b. inside the chamber is not increased if "on demand, built-in-breathing oxygen masks" (BIBS) are used with virus filters on the exhalation hose. If "hoods" or "high flow- masks" are used there is a significantly higher risk for viral contamination if it leaks or is accidentally removed.
   c. should be known by attending staff that need to wear protective gear according to local guidelines.
2. The risk of deterioration in gas exchange:
   a. During HBO the alveolar partial pressure of oxygen (PAO_2) is = 228.4 kPa (PAO_2:240 - PAH_2O:6.3 - $PACO_2$:5.3). The risk of deterioration in oxygenation during HBO is negligible, but a transient decline in arterial oxygenation (PaO_2) has been seen in intubated patients the first few hours after HBO. Safety checks of SpO_2 (and PO_2/PCO_2 if warranted) 1h and 6h post HBO is part of the protocol.
   b. There is a risk is carbon dioxide (CO_2) retention due to increased work of breathing. Therefore, a clinical assessment of work of breathing, including arterial SpO_2, PO_2 and PCO_2, if warranted, is part of the protocol at -1h before HBO.
3. The risk of SAE during and immediately after treatment:
   a. Staff attending the patients should be trained to manage situations such as need for intubation, circulatory chock, cardiac arrest and pneumothorax (according to local guidelines).

Monitoring will be conducted at each trial site before, during and after the trial according to the monitoring plan. Interim analysis for safety and efficacy will be conducted after 20, and 70 subjects.

In summary, we believe the benefits for subjects, the risk-group and public health well outweigh the risks.

4. Study objectives

The overall hypothesis to be evaluated is that HBO reduces mortality, increases hypoxia tolerance and prevents organ failure in patients with COVID19 pneumonitis by attenuating the inflammatory response.

4.1 Primary objective

To evaluate if HBO reduces the number of ICU admissions compared to Best practice for COVID-19.

4.2 Secondary objective(s)

4.2.1 Main secondary objective

To evaluate if HBO:

- reduces mortality in severe cases of COVID-19.
- reduces morbidity associated with COVID-19.
- reduces the load on ICU resources in COVID-19.
- mitigates the inflammatory reaction in COVID-19.

4.2.2 Other secondary objectives

- Investigate how CPTU correlates with outcome in COVID-19.
- Investigate how changes in inflammatory profile in blood correlate with disease severity and outcome.
- Investigate how changes in vital parameters and PFI correlate with outcome
- Investigate if HBO reduces pulmonary edema, and Inflammatory Macrophage activity in SARS-CoV-2 positive patients.
- Explore HBO mechanisms including several inflammatory pathways that can be monitored in blood and plasma.
- Explore how changes in expression of HIF 1-3 regulated genes in PBMC correlate with disease severity and outcome (cohort of 20 subjects).
- Explore how changes in Plasma MicroRNA interacting with HIF 1-3 regulated genes correlate with disease severity and outcome (cohort of 20 subjects).
- Evaluate microRNA as potential biomarkers for outcome.
- Evaluate if HBO is safe for SARS-CoV-2 positive patients and staff.

4.3 Primary endpoint:

The proportion of subjects admitted to or selected for ICU (including ECMO) from day 1 to day 30, based on at least one of the following criteria at the discretion of the investigator:

i) Rapid progression over hours.
ii) Lack of improvement on high flow oxygen >40L/min or non-invasive ventilation with fraction of inspired oxygen (FiO_2) > 0.6.
iii) Evolving Hypercapnia or increased work of breathing not responding to increased oxygen despite maximum standard of care available outside ICU.

iv) Hemodynamic instability or multi organ failure with maximum standard of care available outside ICU.

4.4 Secondary endpoints:

4.4.1 Secondary Efficacy Endpoints

4.4.1.1 Main Secondary Efficacy Endpoints

I. Proportion of subjects with 30-day mortality, all-cause mortality, from day 1 to day 30.

II. Time-to-Intubation, i.e. cumulative days free of invasive mechanical ventilation, from day 1 to day 30.

III. Time-to-ICU, i.e. cumulative ICU free days, derived as the number of days from day 1 to ICU, where all ICU free subjects are censored at day 30.

IV. Mean change in inflammatory response from day 1 to day 30.
   a. White cell count + differentiation
   b. Procalcitonin
   c. C-Reactive protein
   d. Cytokines (IL-6) (if available at local laboratory)
   e. Ferritin
   f. D-Dimer
   g. LDH

VI. Overall Survival.

4.4.1.2 Other Efficacy Endpoints

I. Hospital mortality of any cause, proportion of subjects, from day 1 to day 30.

II. ICU mortality, mortality of any cause in ICU, proportion of subjects, from day 1 to day 30.

III. Time-to-stop of intubation/invasive mechanical ventilation, from ICU admission to day 30.

IV. Mean daily NEWS from day 1 to day 30.

V. Mean change in PaO_2/FiO_2 (PFI), from day 1 to day 2, … to day 30.

VI. HBO Compliance.
   a. Proportion of HBO treatments given vs planned.
   b. Proportion of subjects with HBO treatment administered within 24h after enrolment.

VII. Time-to-discharge from hospital.

4.4.2 Exploratory/Descriptive Endpoints

I. Mean oxygen dose per day including HBO and cumulative pulmonary oxygen toxicity expressed as Units of oxygen pulmonary toxicity dose (UPTD) and Cumulative pulmonary toxicity dose (CPTD) from day 1 to day 30.

II. Median number of HBO treatments and dose of HBO given, from day 1 to day 7.

III. Change in expression of Micro RNA in plasma from day 1 to day 30.
IV. Change in gene expression and Micro RNA interactions in Peripheral Blood Mononuclear Cells (PBMC) from day 1 to day 30.
V. Immunological response (20 subjects) from day 1 to day 30 in the following.
   a. Cytokines extended including (IL-1β, IL-2, IL-6, IL33 and TNFα)
   b. Lymphocyte profile
   c. Flowcytometry with identification of monocyte/lymphocyte subsets including but not limited to CD3+/CD4+/CD8+ and CD4+/CD8+ ratio
   d. FITMaN panel/Flow cytometry, Interleukins (IL-1β, IL-2, IL-6, IL33 and TNFα),
   e. T-reg cells (CD3+/CD4+/CD25+/CD127+)
   f. Monocyte proliferation markers, Ex vivo monocyte function
VI. Mean change in routine biomarkers for organ dysfunction, from day 1 to day 30.
VII. Viral load, from day 1 to day 30.
VIII. Number of secondary infections, number of events and patients from day 1 to day 30.
IX. Diagnosed PE needing treatment, number of events and patients from day 30.
X. Changes on Pulmonary CT from day 1 to day 30.
XI. Changes on Chest X-ray, from day 1 to day 30.
XII. Changes in Lung ultrasound, from day 1 to day 30.

4.4.3 Safety Endpoints

I. Number of subjects, proportion of subjects and number of events of AE.
II. Number of subjects, proportion of subjects and number of events of SAE.
III. Number of subjects, proportion of subjects and number of events of SADR.
IV. Mean change in PaO_2/FiO_2 before and after HBO compared to mean variance in PaO_2/FiO_2 in control group during day 1 to day 7.
V. Mean change in NEWS before and after HBO compared to mean change in daily NEWS in control group during day 1-day 7.
VI. Number of negative events in staff associated with treatment of subject, (e.g. contact with aerosol from subject), number of events from day 1 to day 30 or last day in hospital if subject is discharged earlier, or at withdrawal.

5. Study design and procedures

5.1 Overall Study design

Phase II Clinical Trial

Prospective randomized, open label, multi-centre trial with an estimated enrolment of 200 subjects (20+180). The randomization procedure is described in section 7.5.

Parallel group

Intervention: Hyperbaric oxygen (HBO) in addition to best practice compared with best practice

Study Code: COVID-19-HBO
Version No: v.4
Date: 2021-02-27
EudraCT No: 2020-001349-37

HBO: HBO 1.6-2.4 ATA for 30-60 min, maximum five treatments within seven days from inclusion.

Control: Best practice for COVID-19 pneumonitis.

The first HBO treatment will be given within 24 hours after inclusion. Patients with respiratory symptoms admitted to the hospital will be informed and asked to participate. The patients will be included once they fulfil the inclusion criteria and none of the exclusion criteria, but the timing of the HBO treatment will depend on available resources.

Due to the nature of the epidemic, the available resources and the risk of transport and contamination, it would be unethical and possibly unsafe to conduct a placebo-controlled trial. In the evaluation of safety and efficacy this will be considered.

Clinical equipoise: The rationale for 1:1 randomization is that COVID-19 infection is a new disease and we will use a slightly lower dose of HBO than often used in more stable patients without acute lung injury. Furthermore, 1:1 allocation will maximise the statistical power. If the interim analysis can show supportive evidence for efficacy the trial committee/safety and data monitoring board may choose to change the randomization to 2:1.

In 20 subjects at Karolinska University hospital, extended explanatory immunology/genomic data will be collected. These subjects will be recruited from one specific site that has the ability to perform the analyses.

The trial continues for 30 days after inclusion or until withdrawal.

5.2 Procedures and flow chart

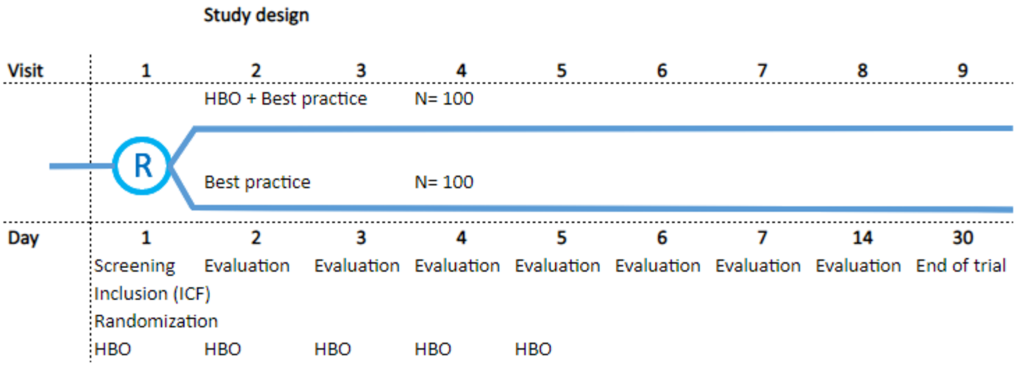

Treatments can be distributed differently depending on clinical effect and availible resources.

5.2.1 Study schedule

Each visit consists of 3 parts:

a) Review of medical records since last visit and documentation in the eCRF.
b) Measurements and actions to correct any deviations.
c) HBO Treatment (Visit 1-7 only).

Study Code:	COVID-19-HBO
Version No:	v.4
Date:	2021-02-27
EudraCT No:	2020-001349-37

Visit 1: (Day 1)

a) After the patient has been informed about the study and agreed to participate, an **informed consent** form (ICF) will be **signed** before any study specific procedures occur. During the **Screening**, procedures to assure the patient´s eligibility for the study participation will be performed such as a baseline ABG sample (if not available for the same day, after 8am, from the patient's medical records) and serum pregnancy test in female subjects of childbearing potential.; **Demographics**, **medical history** including COVID-19 specific history, routine blood tests, secondary infections, viral load and radiology will be reviewed. Concomitant **medications** including oxygen dose (CPTD) since admission, before inclusion will be recorded. **Mean NEWS for the past 24 hours (3 measurements 08, 14, 22 +/-3h)** will be recorded if available (mean is calculated after data is exported from eCRF at the end of Study). Baseline NEWS at inclusion will also be recorded unless it coincides with any of the three timepoints scheduled for NEWS described in section b below. A **physical examination** will be performed and a **HBO specific** questionary as per local routine will be obtained. Subject will then be **randomized** to either HBO (in addition to best practice) or best practice.

b) Non-fasting **blood samples** will be collected, **routine chemistry** will be checked, recorded and if necessary supplemented. **Study specific blood** tests and blood/plasma for future biomedical research will be collected and time shall be recorded. NEWS will be collected three times during 24h, **8am (08:00), 2pm (14:00)**, **10pm (22:00) (+/-3h)**. PFI (collected from ABG/CBG) will be confirmed at least once after inclusion, additional if warranted. The number of **NEWS** depends on when, during the day, the subject is included in the study.

If the subject is randomized to **HBO additional NEWS** should be recorded **-1hour (+/- 45 min)** prior to HBO treatment, **+1hour (+/- 45 min)** after HBO treatment and **6h (+/- 2 hours)** after HBO treatment (marked as † and ‡ in the list of procedures) unless it coincides with routine NEWS. **NEWS (and ABG/CBG,** if warranted), shall be checked by an investigator and if **any deviation**, **action** shall be taken and/or **reported** to the ward physician for **both groups**.

c) Subject will be **transported to the hyperbaric chamber** and given **HBO within 24 hours from randomization**, time and date are recorded. If planned but not given, this should be recorded, including the reason for not giving the treatment.

Visit 2-7: (Day 2-7)

a) **Review of medical records** for changes in concomitant medication, DNR status, routine blood tests, AE, secondary infections, viral load, radiology and review of data on Staff Safety. Documentation of Cumulative Oxygen dose (**UPTD)** previous 24 hours. **NEWS** previous 24 hours **(3 measurements 08, 14, 22 +/- 3h)** (Mean is calculated after data is exported from eCRF at the end of study).

b) **Routine** and **study specific blood** tests including CBG/ABG at **8 am (+/- 3 h).**
If the subject is considered unstable in SpO_2 or has increased work of breathing, CBG/ABG will be collected and **NEWS** are performed and documented at the same time.
In subjects randomized to **HBO, NEWS** will be taken and recorded **-1hour (+/- 45 min)** prior to HBO treatment, **+1hour (+/- 45 min)** after HBO treatment and **6h (+/- 2hours)** after HBO

treatment (marked as † and ‡ in the list of procedures) until the subject is considered to be “in stable condition” by the ward physishian. CBG/ABG shall be collected and analyzed upon clinical deterioration, suspicion of CO_2-retention or increase in NEWS. NEWS and CBG/**ABGs** shall be checked by an investigator and if **any deviation**, **action** shall be taken and/or **reported** to the ward physician for **both groups**.
c) Subject will be **transported to the hyperbaric chamber** and given **HBO (maximum 5 of the first 7 visits)**, time and date are recorded. If planned but not given, this will be recorded including the reason for not giving the treatment.

Visit 8 and 9: (Day 14 and Day 30)

a) **Review of medical records** since previous visit 8:00 (8am) to 7:59 (7:59am) for changes in concomitant medication, DNR status, routine blood tests, AE (e.g. ICU admission, Intubation, secondary infections), viral load, radiology and review of data on Staff Safety. If subject is still admitted to hospital; Documentation of Cumulative Oxygen dose (**UPTD)** previous week for visit 8 and previous 2 weeks for visit 9. **NEWS** previous week **(maximum three measurements 08, 14, 22 +/-3h** (Mean is calculated after data is exported from eCRF at the end of study). At visit 9, medical records will be reviewed for changes in concomitant medication, DNR status, routine blood tests, AE (e.g. ICU admission, Intubation), secondary infections, viral load, radiology and review of data on Staff Safety, until end of visit 9 (i.e. end of study)

b) **Routine** and **study specific blood** tests at **8 am (+/- 3 h).**
If the subject is still in hospital and considered to be “in stable condition” by the ward physishian, CBG/ABG will be taken on clinical deterioration, suspiscion of CO_2 retention or increase in NEWS.
NEWS are performed and documented minimum once, maximum three times during 24h, **8am (08:00), 2pm (14:00)**, **10pm (22:00) (+/-3h)** and **CBG/ABG** will be collected if clinically warranted at the same time points. Routine and study specific blood tests will be collected in hospitalized patients and 20 patients in the Karolinska subgroup regardless of hospital admission.

End of Study

A final visit in the electronic case report form (eCRF) should be completed for every randomised patient whether the patient completed the study or not. The reason for any early discontinuation should be indicated on this form.

5.2.2 Assessments and procedures

Medical history

Relevant medical history such as risk factors in this trial and any other disease affecting the immune system, respiratory or circulatory systems will be recorded at Visit 1. The medical history will include a review of past and current relevant diseases/diagnoses/symptoms. Diagnosis/symptoms/signs during and the start year (of diagnosis) will be collected. A specific evaluation/medical exam will be focusing on HBO specific relative contraindications according

to local routines. Findings and/or abnormalities detected will be recorded in the eCRF. Other medical history, not relevant for the trial will be documented in medical records.

Demography

Demographic data such as gender, age, race, body weight, height, restrictions in escalation of care e.g. DNR and smoking habits will be collected at Visit 1. Records will be reviewed for update/change in DNR status at each visit.

Concomitant medication

Information regarding prior and concomitant medications will be collected at Visit 1. The Investigator or designee will assess changes in concomitant medications e. g. stop date or entry of a new treatment, throughout the study by reviewing the patient's medical records. Any changes will be recorded in the electronic Case Report Form (eCRF).

NEWS SOP

NEWS chart will be assessed as mean NEWS during 24 hours, NEWS will be assessed at 8am (08:00), 2pm (14:00), 10pm (22:00) (+/-3h). Mean NEWS during 24 hours is calculated from exported eCRF data.

Resp Rate (RPM), SpO_2, Supplemental oxygen Y/N, Temperature (deg C), Heart Rate (BPM), Systolic Blood Pressure, Consciousness (VPU). The number of NEWS assesements will depend on the subject's condition and shall be recorded daily as long as the patient is admitted to hospital.

National Early Warning Score (NEWS)*

PHYSIOLOGICAL PARAMETERS	3	2	1	0	1	2	3
Respiration Rate	≤8		9 - 11	12 - 20		21 - 24	≥25
Oxygen Saturations	≤91	92 - 93	94 - 95	≥96			
Any Supplemental Oxygen		Yes		No			
Temperature	≤35.0		35.1 - 36.0	36.1 - 38.0	38.1 - 39.0	≥39.1	
Systolic BP	≤90	91 - 100	101 - 110	111 - 219			≥220
Heart Rate	≤40		41 - 50	51 - 90	91 - 110	111 - 130	≥131
Level of Consciousness				A			V, P, or U

*The NEWS initiative flowed from the Royal College of Physicians' NEWS Development and Implementation Group (NEWSDIG) report, and was jointly developed and funded in collaboration with the Royal College of Physicians, Royal College of Nursing, National Outreach Forum and NHS Training for Innovation

Please see next page for explanatory text about this chart.

Royal College of Physicians

NHS Training for Innovation

Blood samples

All details regarding the blood sampling for all laboratory analysis will be provided in the Laboratory Manual.

HBO SOP and assessment

Each site will have their own SOP according to their local guidelines but in general terms:

Patients will be transported from the ward to the multiplace or monoplace chamber depending on severity on inclusion (according to local guidelines). Patients will be treated with 30-60 min HBO (1.6-2.4 Bar with 5-15 min compression time and 5-15 minutes decompression time, according to local routines). The number of treatments and timing will depend on available resources and clinical efficacy at the discretion of the attending physician. If the patient does not respond in any way to 30 min the first day, the attending physician may choose to treat the patient for 60 minutes instead of 30min. HBO treatment will likely stop if the patient is intubated or admitted to the ICU. However if any site has the resources to continue HBO from the ICU, it may continue; if the subject is intubated, the ventilator should ideally not be changed and if necessary, the endotracheal tube should be clamped to maintain the positive end-expiratory pressure (PEEP) and prevent risk of viral spread.

Date and time for administered HBO treatment will be recorded. HBO treatment that was planned but could not be administered, including the reason, will be recorded.

AE and ADR

Adverse events and collection of Adverse Events and Serious Adverse Events

Collection of AE will start directly after inclusion. Definitions, documentation and reporting of AEs are described in detail in AE section below.

UPTD calculation (not mandatory)

For practical reasons the ambient air pressure one atmosphere will be estimated to 1 ATA. Review of records, the mean oxygen dose at 3 time timepoint will be calculated 8am, 2pm, 10pm +/-2 hours (for baseline calculated since admission) Calculate daily UPTD past 24 hours, Mean FiO_2x 60 x 24 = CPTD (for baseline calculated since admission).

1 UPTD is equivalent of breathing 100% oxygen at 1 atmosphere for 1 minute. E.g. 100% oxygen for 24 hours equals: 1.0 ATA x 60 min x 24 hours = 1440 UPTD

The following conversion table will be used to estimate UPTD

CPTD will be calculated as the total UPTD received during trial, calculation will be done after data is exported from eCRF at the end of study.

.	Type	L/min	O_2	UPTD /24h
High flow nasal or CPAP recorded as % administered	CPAP	10-50	100%	1440
	Reservoir	12-15	80%	1152
If Hudson mask or nasal piece is used convert L/m to the following	Reservoir	10	65%	936
	Hudson	9	60%	864
	Hudson/Reservoir	8	55%	792
	Hudson	7	48%	691
	Hudson	6	44%	633
	Hudson	5	40%	576
	Nasal prongs	4	33%	475

Nasal prongs	3	30%	432
Nasal prongs	2	27%	390
Nasal prongs	1	24%	346

ICU admission

Review of records and documented time of ICU admission and reason for admission, if/when discharge documented time and reason.

Intubation

Review of records and documented time when the subject was intubated, reason for intubation/ invasive ventilation and time for extubation. If patient is tracheostomized, this will be noted but will be regarded as intubation. If tracheostomized, 24 hours without mechanical ventilation will be time regarded as time for stop of invasive ventilation.

ICU mortality

Review of records and documented time and cause of death.

Hospital mortality

Review of records and documented time and cause of death.

Overall mortality

Review of records, documentation, dead or alive at end of study.

Secondary infections

Review of records and document: time of diagnosis, site of infection, classified as suspected or confirmed, microorganism if known (confirmed).

Viral load

Review of records and documented time and result from quantitative PCR.

Staff safety

Review of hospital incidence reports, documented time and a detailed description of the event.

Change on Pulmonary CT

Review records and document time of radiology, reason for radiology, finding. Baseline (first radiology) Categorized as mild, moderate, severe. Change from last radiology classified as improvement, deterioration or no change.

Change on Chest X-ray

Review records and document time of radiology, reason for radiology, finding. Baseline (first radiology) Categorized as mild, moderate, severe. Change from last radiology classified as improvement, deterioration or no change.

Study Code: COVID-19-HBO
Version No: v.4
Date: 2021-02-27
EudraCT No: 2020-001349-37

Lung Ultrasound (LUS) SOP and assessment

When possible, patients will be assessed with transthoracic Ultrasound to evaluate atelectasis/consolidation and pulmonary edema according to a formalized protocol Bedside Lung Ultrasound in Emergency (BLUE), which have a sensitivity and specificity of 93% respectively for interstitial syndrome (Lichtenstein, 2014). These are marked as (LUS) in the procedure list and will be marked in the eCRF if performed. 3 or more B-lines “Lung rockets” in one intercostal space will be regarded as “interstitial syndrome”. Photo or film must be saved to a usb stick or to the hospitals database in order to validate the data.

Review records and document time of LUS, reason for LUS, finding. Baseline (first LUS) Categorized as interstitial syndrome or no interstitial syndrome. Change from last LUS is classified as improvement, deterioration or no change.

Table 1. **List of procedures**

Visit 1-7 is 08-07:59 and Visit 8 and 9 are 7 days 08-07:59

Activity	Visit 1	Visit 2	Visit 3	Visit 4	Visit 5	Visit 6	Visit 7	Visit 8	Visit 9
Day	Day 1	Day 2	Day 3	Day 4	Day 5	Day 6	Day 7	Day 14	Day30
Screening	x								
Inclusion/excl criteria	x								
Pregnancy test if woman of childbearing age	x								
HBO specific medical history/physical examination	x								
Signed Informed consent Form#	x								
Randomization	x								
1. Medical history	x								
2. Demography*	x	x	x	x	x	x	x	x	x
3. Concomitant medications	x	x	x	x	x	x	x	x	x
4. NEWS score	x, x, x**	x, x, x	x, x, x	x, x, x	x, x, x	x, x, x	x, x, x	x, x, x	x, x, x
5. Standard/ study specific biochemistry	x	x	x	x	x	x	x	x	x
6. Study specific CBG/ABG***	x	x	x	x	x	x	x	x	x
7. Plasma (microRNA)	x	x	x	x	x	x	x	x	x
8. HBO specific NEWS/ CBG/ABG †‡	3x?	3x?	3x?	3x?	3x?	3x?	3x?		

Kjellberg A, *et al. BMJ Open* 2021; 11:e046738. doi: 10.1136/bmjopen-2020-046738

Study Code: COVID-19-HBO
Version No: v.4
Date: 2021-02-27
EudraCT No: 2020-001349-37

9. HBO indicated/planned	x	x	x	x	x	x	x		
10. HBO treatment	x	x	x	x	x	x	x		
11. AE	x	x	x	x	x	x	x	x	x
12. ADR	x	x	x	x	x	x	x	x	x
13. UPTD	x	x	x	x	x	x	x	x	x
14. CPTD	x								x
15. ICU admission		x	x	x	x	x	x	x	x
16. Intubation/ mechanical ventilation		x	x	x	x	x	x	x	x
17. ICU mortality		x	x	x	x	x	x	x	x
18. Hospital mortality		x	x	x	x	x	x	x	x
19. Overall mortality		x	x	x	x	x	x	x	x
20. Secondary infections	x	x	x	x	x	x	x	x	x
21. Viral load	x	x	x	x	x	x	x	x	x
22. Staff safety (Negative events)	x	x	x	x	x	x	x	x	x
23. Pulmonary CT (check records)	x	x	x	x	x	x	x	x	x
24. Chest X-ray (check records)	x	x	x	x	x	x	x	x	x
25. Chest Ultrasound (if available)	x	x	x	x	x	x	x	x	x
26. Extended immunology (n=20)	x			x			x	x	x

ICF can be obtained before visit 1*

* Vistit 2-9 Demography check only involves change in DNR status.

** Depending on time of inclusion 1-3 samples/observations will be collected during visit 1 at the specified time points. Additionally, a baseline ABG (if not available from the patient´s medical records) and a baseline NEWS is collected.

*** Once daily at 8am, additional samples if warranted at the discretion of ward physician

†‡ Explaned in detail Section 5.2.1, Visit 1 and 2-7, part b

? HBO specific NEWS (and CBG/ABG if warranted) is only collected on days of HBO treatment.

5.3 Biological sampling procedures

5.3.1 Handling, storage, and destruction of biological samples

Standard biochemistry for kidney function, liver function, cardiac insult (TnT or TnI), haematology and blood glucose will be collected from the hospitals electronic system and entered into the e-CRF.

Laboratory safety assessment /arterial blood gas: will be analysed in local accredited laboratories close to the patients within 15 minutes (Point of Care). Print-outs must be marked with Visit, serial number (-1h, 1h, 6h, 8am, 2pm, 10pm), subject study code, date, time and signed by the investigator. In some centres routine ABG are not collected, then capillary blood gas (CBG) will be accepted for measuring change in PO_2/PCO_2. Inclusion criteria must be based on ABG. Each visit will include 3x1.5ml and additional 3x1.5ml CBG/ABG for HBO during days of treatment. If CBG/ABG is taken as part of routine care at the stated time points, no additional CBG/ABG is necessary.

Study specific blood samples: Interleukin-6 (if available), Procalcitonin, HbA1C (visit1 only), insulin, Ferritin and D-Dimer will be analysed together with routine biochemistry at the accredited local lab, (applicable for sites where analyses are available locally), for most laboratories no additional blood is needed. One EDTA plasma will be bio-banked for later analysis of microRNA in plasma (if possible).

Extended immunology blood samples for 20 patients (explanatory): 2x4ml Citrate CPT-tubes for PBMC isolation, 2x4ml EDTA-tubes for extended lymphocyte analysis.

CPT-tubes will be collected by one of the investigators and transported immediately to the research laboratory where PBMCs are isolated, half are prepared with RNA-later® for later DNA/RNA extraction and gene expression analysis and the other half is cryopreserved for later functional analysis of the monocytes. The monocytes and EDTA plasma will be stored in a sub-biobank at Bioclinicum Karolinska University Hospital. The biological samples will be saved until all analyses are performed.

5.3.2 Total volume of blood per subject

Since most of the blood taken are routine samples for COVID-19, only maximum additional 16 ml (8 ml for all and additionally 8ml for 20 subjects) will be collected. The ABG will depend on the number of HBO treatments, 4.5ml/ treatment, maximum 22.5 ml, if five HBO treatments are given. Maximum 105 ml blood is collected if five HBO treatments are given, for control group 85ml blood. This needs to be related to routine blood samples taken in these critically ill patients that is normally 16-28 ml/day, 480-840 ml over 30 days.

5.3.3 Biobank

Study specific EDTA plasma and PBMC collected in Sweden in this study are released to *Karolinska Institute Biobank (IVO reg. no 222)* and handled according to the current biobank laws and regulations. A national agreement is approved by *Regionalt biobankscentrum Stockholm-Gotland*. The samples are coded/pseudonymized to protect the subject´s

identification. All samples and the identification/code list are stored securely and separately to prevent unauthorized persons from having access to them.

5.3 End of Study

The end of study is defined as the last participant's last follow up.

Premature termination of this clinical study may occur because of a regulatory authority decision or at the discretion of the sponsor.

The sponsor reserves the right to discontinue the study at any time point in the trial in the following cases:
• Unexpected high proportion of AE: s that are possibly or probably related to the study drug.
• Study protocol is difficult to cope with.
• Recruitment of eligible subjects is far too low.

Criteria for premature termination are strict and follow the Haybittle-Peto recommendation with a statistical significance of $p<0.001$.

The end of the study will be reported to the regulatory authority within 90 days, or 15 days if the study is terminated prematurely. The Investigators will inform participants and ensure that the appropriate follow up is arranged for all involved.

6. Subject selection

6.1 Inclusion criteria:

To be included in the study, subjects must meet the following criteria:

1) Aged 18-90 years
2) PaO_2/FiO_2 (PFI) below 200 mmHg (26.7 kPa) (Based on ABG measurement), assessed if (~5L oxygen/min to reach 90% SpO_2)
3) Suspected or verified SARS-CoV-2 infection
4) At least two risk factors for increased morbidity/mortality
   - Age above 50 years
   - Hypertension
   - Cardiovascular disease
   - Diabetes or pre-diabetes
   - Active or cured cancer
   - Asthma/COPD
   - Smoking
   - D-Dimer > 1.0
   - Auto-immune disease
5) Documented informed consent according to ICH-GCP and national regulations

6.2 Exclusion criteria:

Subjects must not be included in the study if any of the following criteria are met:

1) ARDS/pneumonia caused by other viral infections (positive for other virus).
2) ARDS/pneumonia caused by other non-viral infections or trauma.
3) Known pregnancy or positive pregnancy test in women of childbearing age.
4) Patients with previous lung fibrosis more than 10% (verified by CT).
5) CT- or Spirometry-verified severe COPD with Emphysema.
6) Contraindication for HBO according to local guidelines.
7) Not likely to need ICU admission within 7 days of screening (Subjective criteria that may exclude any patients that fulfil the other inclusion criteria but where the treating physician suspect a spontaneous recovery).
8) Mental inability, reluctance or language difficulties that result in difficulty understanding the meaning of study participation.
9) Prisoners.
10) Unable/risk to move patients to hyperbaric chamber.

6.3 Screening

Patients with respiratory symptoms admitted to the hospital will be pre-screened by ward physicians or study officials. Subjects will be informed in detail about the trial by an investigator. After obtaining a written informed consent, additional medical record review, physical examination and (pregnancy test if applicable) will be conducted. Subject eligibility (that subjects fulfill all inclusion criteria and do not meet any exclusion criteria) is established before randomization to treatment.

6.4 Withdrawal Criteria

Patient participation

A patient will be considered to have completed the study when he or she completes the assessment at day 30. Patients should be encouraged to complete the study but have the right to make a decision regarding study participation e.g. to discontinue the study treatment, but still come on visits or discontinue study drug and not come on further study visits. The patient has no obligation to explain why he/she does not want to continue. The investigator has the right to stop the patient's treatment in the event of AE, protocol deviations, administrative reasons or other reasons. It is understood by all concerned that an excessive rate of discontinues can render the study un-interpretable. Therefore, unnecessary discontinuation should be avoided.

Irrespective of the reason for not continuing in the study and whenever possible, the patient should be examined. Relevant laboratory test samples should be obtained and all relevant assessments should be completed, if applicable.

All AEs should be followed up until they have returned to baseline status or stabilised.

A final visit in the electronic case report form (eCRF) should be completed for every randomised patient, whether the patient completed the study or not. The reason for any early discontinuation should be indicated on this form.

Patients may be discontinued from the study at the discretion of the Investigator. Specific reasons for discontinuing a patient from further assessments are:

- AE: Clinical or laboratory events that in the judgment of the investigator, Data Safety Monitoring Board (DSMB) or the Sponsor and in the best interest of the patient constitute grounds for discontinuation. This includes serious and non-serious AE regardless of relation to study drug.
- Withdrawal of Consent: If a patient withdraws consent for disclosure of future information at the discontinuation of the study or after completion of the study, no further evaluations should be performed and no additional data should be collected. The Sponsor may retain and continue to use data collected before patient withdrew his/her consent. The Withdraw Consent reason is only applicable if the patient denies any further contact with site and no further data collection.
- Lack of Efficacy/Treatment Failure: Patients experiencing deterioration or no improvement regarding symptoms, as judged by the investigator, may be discontinued from the study at any time during the study, offered alternative treatment and scored as treatment failures. Treatment failures include disease worsening, requirement for rescue medication for treatment of UC, requirement for surgical intervention and study drug related AE. Patients may be discontinued for sustained non-response at the discretion of investigator.
- Protocol Violation: The patient’s findings, or conduct, fails to meet protocol entry criteria or fails to adhere to the protocol requirements making it impossible to derive sound scientific or medical conclusions from the primary endpoint data generated on a subject, (e.g. Failure to give first HBO treatment within 24h of Randomization).
- Lost to Follow-Up: The patient does not show up for further visits and study personnel can´t reach the patient.
- If the subject is tested negative for SARS-CoV-2 after randomization and no previous positive test that can explain the symtoms is availible, the subject is withdrawn.
- Other: Termination of other reason

If the subject discontinues the study, follow-up of this subject is to be performed according to the clinic’s routine and will be included in the Safety population, if he/she had received at least one treatment.

7. Study treatments

7.1 Description of investigational product(s)

Oxygen 100%, Medical grade

7.2 Dose and administration

Hyperbaric oxygen 1.6-2.4 ATA for 30-60 minutes (with 5-15 min compression time and 5-15 minutes decompression time, according to local routines). The number of treatments and timing will depend on available resources and clinical efficacy at the discretion of the attending physician, with the recommended starting dose being 30 minutes at 2.4 ATA. If the patient does not respond in any way to 30 min the first day, depending on available resources, the attending physician may choose to increase the duration from day 2. The profile recommended by the Sponsor is 2.4 Bar: 60min including 5 min airbrake: 10 min compression/ decompression). No treatment must be given after day 7 (Visit 7), maximum 5 treatments can be given during the first 7 days.

7.3 Packaging, labeling, and handling of investigational products(s)

Compressed from tanks marked 100% Oxygen for medical use or cryogenic gas from hospital supply system depending on local routines. There will be no study specific packaging or labeling.

7.4 Drug accountability and treatment compliance

HBO is delivered inside a hyperbaric chamber by inhaling 100% oxygen through a tight facemask attended by medical staff. If the mask is tight the inspired oxygen pressure is 234,7-240kPa (range depending on 100% saturated - dry gas) at 240 kPa pressure, hence there is no uncertainty about compliance. During compression/decompression patients my need to remove the mask in order to equalize the middle ears and the time might differ according to local protocols. The difference in dose during this period is therefore not counted into the treatment time. If there is no obvious effect of 30 minutes after the first treatment, the attending physician may extend the duration from session 2. That would include five minutes of breathing air för each 30 minutes at pressure. The time of treatment will be recorded in the eCRF.

7.5 Randomization

Subjects will be enrolled and randomized consecutively as they are found to be eligible for inclusion in the study. HBO treatment will start within 24 hours of randomization.

If a subject discontinues their study participation, their subject code will not be reused, and the subject will not be allowed to re-enter the study again. There will be no replacement for these subjects.

Eligible subjects will be randomized in a 1:1 allocation, stratified by site and gender in blocks (blinded to all but the randomizing CRA) to either HBO or Control. There will be a computer generated randomization.

This is an open-label study where patients and investigator will not be blinded to study treatment.

7.6 Concomitant Medication

Medications that are considered necessary for the safety and well-being of the subject can be given at the discretion of the investigator, unless otherwise specified as an exclusion criterion.

All medications that the patient is prescribed and has taken during the study must be recorded in the eCRF. Any changes need to be reported.

7.7 Treatment after study end

After the first seven days no further HBO treatment is to be administered. However normobaric oxygen administration can continue if needed. The total dose during the study will be recorded until and including day 30. After the study ends, the participants will be treated according to routine clinical praxis.

8. Handling of Adverse Events

8.1 Definitions

8.1.1 Adverse Event (AE)

Adverse Event (AE): Any untoward medical occurrence in a clinical investigation subject administered a medicinal product and, which does not necessarily have a causal relationship with the treatment, can be an unfavorable and unintended sign (including an abnormal laboratory discovery), symptom or disease temporally associated with the use of the medicinal (investigational) product, whether or not related to the medicinal (investigational) product.

8.1.2 Adverse Reaction (AR)

In the new use of a medicinal product, all noxious and unintended reactions to the medicinal product related to any dose should be considered an adverse reaction (AR). The phrase "reaction" to a medicinal product means that the causal relationship between the medicinal product and an adverse event is at least a reasonable possibility, that is the relationship cannot be ruled out.

8.1.3 Serious Adverse Event (SAE)

Serious adverse event (SAE): Any untoward medical occurrence that at any dose:

- results in death
- is life-threatening
- requires inpatient hospitalization or prolongation of existing hospitalization
- results in persistent or significant disability or incapacity
- results in a congenital anomaly/malformation
- regarded as medically important without meeting the above mentioned criteria

Medical and scientific assessment will be made to determine if an event is “serious” and whether it would prompt reporting in other situations, for example important medical events that may not be directly life-threatening or result in death or hospitalization but may compromise the study subject or may require intervention to prevent one of the other results set forth in the definitions above. These should also normally be considered as SAEs.

8.1.4 Suspected Unexpected Serious Adverse Reaction (SUSAR)

SUSAR: A reaction/event that is unexpected, serious, and suspected to be caused by the treatment, i.e. adverse events that are not included in the SPC.

8.2 Assessment of Adverse Events

8.2.1 Assessment of causal relationship

The investigator is responsible for determining whether there is a causal relationship between the AE/SAE and the use of the investigational product.

Those AEs which are suspected of having a relationship to the investigational product will be followed up until the subject has recovered or is well taken care of and on their way to good recovery (see also section 8.4, Follow-up of Adverse Events).

All AEs will be categorized either as related, probably related, possibly related, unlikely related or not related, in accordance with the definitions below:

Related: Clinical event, including abnormal results from laboratory analyses, occurring in a plausible temporal sequence in relation to drug administration. The observed event matches with the known adverse reactions scheme for the drug involved. The event cannot be attributed to underlying disease or other medications.

Probably related: Clinical event, including abnormal results from laboratory analyses, occurring within a reasonable time after administration of the investigational product. The observed event match with the known adverse reactions scheme for the drug involved. It is unlikely attributable to underlying disease or other drugs.

Possibly related: Clinical event, including abnormal results from laboratory analyses, occurring within a reasonable time after administration of the intervention/investigational product. The event could be explained by the investigational product and its emergence is reasonable in relationship with use of the investigational product, but there is insufficient information to determine the relationship. The event could be explained by an underlying disease or other medications.

Unlikely related: Clinical event, including abnormal results from laboratory analyses, with a with a temporal relationship with respect to drug exposure that makes a relationship improbable (but not impossible). The event could be plausibly explained by an underlying disease or other medications.

Not related: Clinical event, including abnormal results from laboratory analyses that do not meet any of the above criteria for relatedness.

8.2.2 Assessment of intensity

Each adverse event shall be classified by an investigator as mild, moderate or severe.

Mild: The adverse event is relatively tolerable and transient in nature but does not affect the subject's normal life.

Moderate: The adverse event causes deterioration of function but is transient. The event can be sufficiently unpleasant and need additional treatment with supplement oxygen and/or non-invasive ventilation.

Severe: The adverse event causes deterioration of function to the extent that the subject needs intubation/ICU admission or is immediately life threatening.

8.2.3 Assessment of seriousness

The investigator is responsible for assessing the seriousness (serious or non-serious). If the incident is considered serious, this should be reported as a serious adverse event (SAE) by the investigator to the sponsor. See also section 8.3.2, Reporting of Serious Adverse Events (SAE).

8.3 Reporting and registration of Adverse Events

At each study visit, adverse events (AE) are registered. Collection of AE will start directly after an Informed Consent is signed and continue up to and including day 30 (Visit 9), which is a minimum of 23 days after the subject has ended their treatment with the investigational product. All AE that occur during the study and which are observed by the investigator/study nurse or reported by the subject will be registered in the eCRF regardless of whether they are related to the investigational product or not. Assessment of causal relationship, severity, and whether the AE is considered to be an SAE or not will be done by the investigator directly in the eCRF. At minimum, for each AE/SAE, a description of the event is recorded (diagnosis/symptom if diagnosis is missing), start and stop times, causal relationship, severity, if the AE is considered to be an SAE or not, measures and outcome.

Due to the clinical course of the disease in COVID-19 the following situations will not be reported as AE/SAE:

- A desaturation that can be solved on the ward with additional oxygen only will not be recorded as an AE.
- Desaturation that is transient and can be solved without involvement of ICU/Emergency outreach including Mobile Intensive Group (MIG) or Medical Emergency Team (MET) will not be considered as an SAE. Any desaturation that need CPAP/NIV will be considered as an AE irrespective of Emergency/ICU involvement unless present at inclusion.
- Any change in routine biochemistry will not be reported as AE
- Change in PaO_2 and $PaCO_2$ on ABG will be reported as an AE only if the change leads to a medically significant increase of oxygen and/ or change from a lower level mode

of oxygen delivery (nasal prongs/Hudson Mask>High flow cannula/Non-invasive ventilation>Invasive ventilation

8.3.1 Reporting of Adverse Events (AE)

All AEs to be reported shall be registered in the eCRF continously.

8.3.2 Reporting of Serious Adverse Events (SAE)

Serious adverse events (SAE) are reported to the sponsor on a special SAE form (included in the eCRF) within 24 hours of the investigator being informed of the SAE.

Follow-up information describing the outcome and handling of the SAE is reported as soon as this information is available.

The sponsor will in a timely manner assess whether the adverse event was expected for the investigational product or not, using the reference safety information. Serious AEs must be collected, registered in the CRFs and an assessment of causality of the SAE should be performed. Also, discontinuations due to AEs will be collected.

8.3.3 Reporting of Suspected Unexpected Serious Adverse Reactions (SUSAR)

Those SAE in Sweden which are assessed by sponsor to be SUSAR are reported via a CIOMS form to the MPA that are submitting the CIOMS report to the to the European Medicines Agency (EudraVigilance database) according to the specified time frames.

SUSAR that are fatal or life-threatening are reported as soon as possible and no later than seven days after the incident has become known to the sponsor. Relevant follow-up information is sent thereafter within an additional eight days. Other SUSAR are reported as soon as possible and no later than 15 days after they have come to the sponsor’s knowledge.

Any SUSAR will also be notified to the EPM by the sponsor.

Information about SUSAR occurring during the study is compiled by the sponsor and sent out to the principal investigator at all participating centers in connection to the event.

SUSARs in other participating countries will be reported to respective CA and EC according to applicable procedures

8.4 Follow-up of Adverse Events

All AEs should be followed up until they have returned to baseline status or stabilized. AEs suspected to have a causal relationship with the study intervention are followed until recovered or until the subject is on good way to recovery

8.5 Safety Report (Development Safety Update Report, DSUR)

During the study period, an annual Development and Safety Update Report (DSUR) will be submitted to the competent authorities and ethics committes in all participating countries.

The report includes a summary of all reported SAEs and SUSARs, a summarized safety assessment for study subjects and information regarding potential updates of the risk-benefit assessment since study approval.

8.6 Procedures in case of emergencies

The sponsor and investigator are obliged to immediately take the urgent safety measures necessary to protect the subjects from immediate danger. Examples of such measures are to temporarily suspend the clinical trial or to introduce supplementary monitoring measures. The sponsor shall inform the applicable competent authorities and ethics committees as soon as possible about the urgent safety measures taken by the investigator or sponsor.

8.7 Reference Safety Information

For reference safety information, reference is given in the SmPC.

9. Statistics

9.1 Statistical Analysis Plan

The principal features of the statistical analysis of the data are described in this section. A more technical and detailed elaboration of the principal features will be written in a separate Statistical Analysis Plan (SAP).

9.1.1 Analysis population

9.1.1.1 Definition of Study Populations

9.1.1.2 Intent-to-Treat Population (Full Analysis Set); All randomized subjects will be included in the Intent-to-Treat (ITT) population.

9.1.1.3 Per-Protocol Population; All randomized subjects with no major protocol violations will be included in the Per Protocol (PP) population. The final decisions regarding the PP population will be taken at the Clean File meeting before the database lock.

9.1.1.4 Safety Population; All randomized subjects will be included in the safety population.

9.2 Statistical analyses

9.2.1 Sample size calculations

Power calculation is challenging in COVID-19 since hospitalization and mortality rates differ enormously between publications and seem to be highly variable between different countries. Mortality rates have been reported as high as 90% in patients developing ARDS in early reports from Wuhan province and more recent reports has reported overall 28-d mortality rates of 61,5% in ICU patients with acute respiratory illness (Yang et al., 2020a) In a recent retrospective cohort study form Wuhan 19% of hospitalized patients needed mechanical ventilation or ECMO, of whom 97% died, SIC! 26% was admitted to the ICU and hospital mortality rate was 28%(Zhou et al., 2020). Mortality rates in ARDS in general are until now decreasing but still very high. A recent systemic overview reported mortality rates since 2010: Overall rates of in-hospital- 45%, ICU- 38% and 28/30-d- 30% (Maca et al., 2017). With our inclusion and exclusion criteria we believe that we can select patients at risk for ICU admission, intubation, morbidity and mortality.

The primary endpoint, ICU admission, is defined by criteria for selection for ICU.

We have assumed that 50% of the subjects will have at least one criteria during the course of the study and we aim to reduce the ICU admission rate by 40%, i.e. to an ICU admission rate of 30%. To achieve 80% power with type-I error rate of 0.05, a sample size of 93 subjects per group is required (two-sided). We plan to enrol 200 subjects into this trial. Interim analyses may decide to re-calculate the sample-size for the trial.

The sample size calculation was done in nQuery version 7.

9.2.2 General statistical methodology

Primary and secondary endpoints will be evaluated using the ITT population and the primary endpoint also using the PP population.

9.2.3 Patient Demographic and Baseline Characteristics

Baseline values and patient characteristics will be presented in tables by group and in total. All continuous variables will be described using standard statistical measures, i.e., number of observations, mean and median value, standard deviation, minimum and maximum value. All categorical variables will be summarised in frequency tables.

9.2.4 Primary Endpoint Analysis

The analysis of the primary endpoint is conducted on the FAS and PPS.

The primary analysis of the primary endpoint will be performed using the Cochran Mantel Haenszel test adjusting for randomisation strata site and gender.

The primary endpoint will be analysed for the proportion of patients with ICU admission using an overall type I error rate of 0.05, using a two-sided test.

The p-value for testing the null hypotheses, no difference between treatment groups, must be less than 0.05 to be considered to have met the primary objective.

There will be no adjustment for multiplicity as there is only one primary endpoint.

9.2.5 Secondary Endpoints Analysis

The same analysis approach used for the primary efficacy endpoint will be applied to the secondary efficacy and exploratory endpoints referred to as a “Proportion endpoints”.

Continuous endpoints such as mean change from baseline will be evaluated using the ANCOVA, including the treatment and stratifying factor as fixed factors and the baseline as a covariate in the model.

The time-to-event endpoints will be presented using the Kaplan-Meier method and the test between treatment groups will be done using the log-rank test.

The p-value for testing the null hypotheses, no difference between treatment groups, must be less than 0.05 to be considered to have met the objective.

No multiplicity adjustments will be made for the exploratory endpoints. Handling of secondary efficacy endpoints will be described in detail in the statistical analysis plan.

All analysis will be done for the FAS population.

9.2.6 Safety analyses

Safety analyses will be performed on the Safety population.

9.2.6.1 Analysis of Adverse Events

The number and percentage of patients reporting AEs, and the number of AEs reported will be presented. The events will be tabulated by system organ class and preferred term. In addition, summaries by relationship to study drug and severity will be presented. SAEs will also be presented in separate tabulations.

Kjellberg A, *et al. BMJ Open* 2021; 11:e046738. doi: 10.1136/bmjopen-2020-046738

The number of patients experiencing an AE will be compared descriptively between groups. All patients with AEs will be listed individually with patient number in addition to type of event, start and stop time, duration, seriousness, severity, action taken, relationship to study drug and outcome of AE.

9.2.6.2 Other Safety Assessments

All continuous safety variables, such as laboratory measurements, vital signs, ECG parameters, and body weight will be described using summary statistics. Changes from baseline will also be summarised as appropriate.

All categorical variables, such as physical examination, will be summarised using frequencies and percentages.

The safety will include laboratory safety variables and/or adverse clinical findings as appropriate. Laboratory data will also be presented in shift tables for selected parameters, where the number of values within, below and above laboratory reference range will be displayed.

Interim analyses will be conducted after 20 and 70 subjects for safety variables, SAE and AE.

9.2.7 Interim Analysis

Safety will be monitored continuously by the safety monitoring board throughout the trial.

There will be an interim analysis performed after 70 subjects have available data for the primary endpoint. The purpose for the interim analysis is to stop for futility if efficacy has not been established. Also, if there is an evidence of a superior efficacy with a delta of 20 % or more, the study will continue with a 2:1 randomisation.

A data and safety monitoring board will perform the interim analysis. A separate DSMB protocol will be created.

9.2.8 Handling of Dropouts and Missing Data

For the efficacy analyses, missing data will in general be replaced using the non-responder imputation (NRI). NRI will be used where missing data are replaced with a negative outcome, i.e. interpreted as a non-responder to the intervention, and sensitivity analysis using alternative methods for replacement of missing data may be considered and will be specified in the statistical analysis plan.

10. Quality Control and Quality Assurance

10.1 Quality Assurance and Sponsor oversight

Steps to be taken to ensure the accuracy and reliability of data include the selection of qualified investigators and appropriate study centers, review of protocol procedures with the site

personnel before the study. eCRF completion guidelines will be provided and reviewed with study personnel before the start of the study.

10.2 Monitoring

The study will be monitored by an independent monitor before the study begins, during the study conduct, and after the study has been completed, so as to ensure that the study is carried out according to the protocol and that data is collected, documented, and reported according to ICH-GCP and applicable ethical and regulatory requirements. Monitoring is performed as per the study's monitoring plan and is intended to ensure that the subject's rights, safety, and well-being are met as well as data in the CRF are complete, correct, and consistent with the source data. The monitoring will be performed by an independent experienced monitor qualified in ICH GCP, applicable national and international regulations and the Declaration of Helsinki.

10.3 Source data

The investigator must keep source documents for each subject in the study. Data in the eCRF can be source data, such as for certain demography parameters, sampling of study specific blood samples and assessment of AEs. A document describing what has been classified as source data in the study should be included in the Investigator Site File (ISF). The investigator must ensure that all source documents are accessible for monitoring and other quality control activities.

Source data is defined before study start at each individual site.

10.4 Deviations or serious breaches

Serious breaches and deviations from the study protocol, GCP and other regulations that significantly and directly affects, or with high likelihood could affect, the subjects in Sweden or the scientific value of the study, shall be immediately reported within 7 days (from knowledge) to the Swedish MPA. It is the sponsor's responsibility to judge the consequences of deviations that have occurred, and thus also to decide whether the Swedish MPA should be informed.

Serious breaches in other participating countries will be reported according to national procedures.

For major protocol deviations i.e violations see also section 6.4.

Minor deviations that do not affect subjects' integrity or safety, nor significantly affect the study's scientific value, are documented in the study documentation of the principal investigator and the sponsor.

10.5 Audits and inspections

Authorized representatives for the sponsor and Competent Authorities (CA) may carry out audits or inspections at the study site, including source data verification. The investigator must

ensure that all source documents are available for audits and inspections. The purpose of an audit or inspection is to systematically and independently review all study-related activities and documents, so as to determine whether these activities were performed, registered, analyzed and reported correctly according to protocol, Good Clinical Practice (GCP) and applicable regulations.

10.6 Data Safety Monitoring Board

An independent DSMB will evaluate the safety data in the context of the overall trial and the currently existing information about the study drug. The DSMB will be composed of representatives from and experts in their respective disciplines of medicine, statistics and clinical trial methodology and conduct.

The DSMB will review the data during the course of the study, as specified in table 2 below, and will draw up a charter delineating their guidelines for operating and stopping rules for terminating individual patients, a portion or all of the trial prematurely. However, the DSMB may for any safety concerns recommend stopping of the trial even if these criteria are not fulfilled. It is the responsibility of the Sponsor to decide whether premature end of study will be made, based on the advice provided by the DSMB.

The DSMB will have access to all trial data. It may request and will be provided with whatever data is deemed necessary or useful for it to carry out its duties. The data provided will be blinded to treatment group unless specific unblinding is requested by the DSMB.

**Table 2. DSMB meeting schedule**	**Time of meeting**
Before study start	Before first subject is included
Safety Interim analysis	When 20 subjects have completed visit 9
Interim analysis	When 70 subjects have completed visit 9
End of the study	Last visit has been done by the last patient.

10.7 Data protection

If any part of the data is handled by any other organization, inside or outside the EU, appropriate agreements and/or other documentation will be established, to ensure that the data processing is performed in accordance with the provisions of the General Data Protection Regulation (GDPR) and other relevant legislation, before any data transfer takes place.

The content of the informed consent form complies with relevant integrity and data protection legislation. In the subject information and the informed consent form, the subject will be given complete information about how collection, use and publication of their study data will take place. The subject information and the informed consent form will explain how study data are stored to maintain confidentiality in accordance with national data legislation. All information processed by the sponsor will be pseudonymized and identified with a Study ID.

The informed consent form will also explain that for verification of the data, authorized representatives of the sponsor, as well as relevant authority, may require access to parts of medical records or study records that are relevant to the study, including the subject's medical history.

11. Ethics

11.1 Compliance to the protocol, GCP and regulations

The study will be performed in compliance with the study protocol, the Declaration of Helsinki, ICH-GCP (Good Clinical Practice) guidelines and current national and international regulations governing this clinical trial. This is to ensure the safety and integrity of the study subjects as well as the quality of the data collected.

11.2 Ethical review of the study

The final study protocol for clinical trials must be approved, as a part of the application for a permit for clinical trials, by both the Swedish Ethical Review Authority (Etikprövningsmyndigheten, EPM) and the Swedish Medical Products Agency (MPA) before the trial can be conducted. The final version of the informed consent form and other information provided to subjects, must be approved or given a written positive opinion by EPM. EPM and the Swedish Medical Products Agency must be informed of any changes in the study protocol in accordance with current requirements. Each trial site outside Sweden must apply for ethical approval by their local ethics comittee and national competent authority and the subject written information and consent form must be provided in the local language.

11.3 Procedure for obtaining informed consent

The principal investigator at each site shall ensure that the subject is given full and adequate oral and written information about the study, its purpose, any risks and benefits as well as inclusion and exclusion criteria. Subjects must also be informed that they are free to discontinue their participation in the study at any time without having to provide a reason. Subjects should be given the opportunity to ask questions and be allowed time to consider the provided information. If the person chooses to participate, both the subject and the investigator shall sign the informed consent form. A copy of the subject information as well as the informed consent form shall be provided to the subject. The subject's signed and dated informed consent must be obtained before performing any study-specific activity in the study. Each subject who participated in the study will be identified by a subject number on a subject identification list. The subject agrees that monitors, auditors, and inspectors may have access to their medical records and other source data. If new information is added to the study, the subject has the right to reconsider whether he/she will continue their participation.

Due to the risk of spreading the infection the consent form needs to be signed by the subject and the investigator inside the room. The signed form will be photographed (scanned), and

the original will stay with the subject. The digital copy will then be printed and contra-signed by the investigator outside the room and then regarded as the original (source data), the original paper will stay with the subject. The digital copy will be destroyed at the latest at the end of the study. The subject can ask for a new copy of the source data once he/she recovers.

12. Insurances

Study subjects are covered by the patient injury insurance and the Swedish pharmaceutical insurance for Swedish sites. Sites outside Sweden must specify what insurance apply in their country/site before any subjects can be enrolled in the study.

13. Substantial changes to the study

Substantial changes to the signed study protocol are only possible through approved protocol amendments and by agreement from all responsible persons. Information on non-substantial changes should be clearly noted in the amended protocol.

In the event that substantial changes to the protocol (e.g., changing of the main objective, primary or secondary variables, method to measure the primary variable, changing of the investigational product or dosage) will be made during the course of the study, approval from the national competent authority and ethics committee shall be obtained before any changes are implemented in that country. A change that concerns a new site, new investigator or a new study patient information sheet shall only be approved by the ethics committee, as applicable.

Non-substantial changes will be recorded and later entered in documentation that is submitted, for example in any subsequent notifications of a substantial change or in connection with End of Trial reporting.

14. Collection, handling and archiving data

Subjects who participate in the study are coded with a specific study identification number. All subjects are registered in a subject identification list (subject enrolment and identification list) that connects the subject’s name and personal identity number with a study identification number.

All data will be registered, managed, and stored in a manner that enables correct reporting, interpretation, and verification. The complete Trial Master File, as well as source documents, will be archived for at least 10 years after the study is completed. Source data in the medical records system is stored and archived in accordance with the respective hospital regulations.

14.1 Case Report Form

An electronic Case Report Form (eCRF) is used for data collection. The investigator must ensure that data is registered and any corrections in the eCRF are made as stated in the study

protocol and in accordance with the instructions. The investigator must ensure that the registered data is correct, complete, and that reporting takes place according to the timelines that have been predefined and agreed. The investigator signs the completed CRF. A copy of the completed CRF will be archived at the study site.

If an examination/test is not performed and data does not exist, ND (Not done) or NK (Not known) is marked. If the question is irrelevant NA (Not applicable) is written. Corrections in paper work sheets are done by striking out the incorrect information and adding the correct information next to the incorrect information, signing, and dating the correction.

15. Notification of study completion, reporting, and publication

The Swedish MPA, EPM, local IRBs, FDA and other national competent authorities and ethics committes shall be informed of the study’s completion at latest 90 days after study end, through submission of a ”Declaration of End of Trial Notification” form.

Within one year after the study is completed, the results shall be analyzed, a clinical study report with individual data shall be prepared, and the study results shall also be reported in the EudraCT database. The sponsor is responsible for the preparation of the clinical study report. The statistical analyses will be performed and the results will be presented to the Investigator(s). Based on these data, the Sponsor, in cooperation with the Investigator(s), will prepare a clinical study report. The report will be submitted to the competent authorities and may form the basis for a manuscript intended for publication in a medical/scientific journal. All personnel who have contributed significantly with the planning and performance of the study may be included in the list of authors.

16. References

ARABI, Y. M., MURTHY, S. & WEBB, S. 2020. COVID-19: a novel coronavirus and a novel challenge for critical care. *Intensive Care Med*.

BENSON, R. M., MINTER, L. M., OSBORNE, B. A. & GRANOWITZ, E. V. 2003. Hyperbaric oxygen inhibits stimulus-induced proinflammatory cytokine synthesis by human blood-derived monocyte-macrophages. *Clin Exp Immunol,* 134**,** 57-62.

BRUNE, B., DEHNE, N., GROSSMANN, N., JUNG, M., NAMGALADZE, D., SCHMID, T., VON KNETHEN, A. & WEIGERT, A. 2013. Redox control of inflammation in macrophages. *Antioxid Redox Signal,* 19**,** 595-637.

BURAS, J. A., HOLT, D., ORLOW, D., BELIKOFF, B., PAVLIDES, S. & REENSTRA, W. R. 2006. Hyperbaric oxygen protects from sepsis mortality via an interleukin-10-dependent mechanism. *Crit Care Med,* 34**,** 2624-9.

CAO, B., WANG, Y., WEN, D., LIU, W., WANG, J., FAN, G., RUAN, L., SONG, B., CAI, Y., WEI, M., LI, X., XIA, J., CHEN, N., XIANG, J., YU, T., BAI, T., XIE, X., ZHANG, L., LI, C., YUAN, Y., CHEN, H., LI, H., HUANG, H., TU, S., GONG, F., LIU, Y., WEI, Y., DONG, C., ZHOU, F., GU, X., XU, J., LIU, Z., ZHANG, Y., LI, H., SHANG, L., WANG, K., LI, K., ZHOU, X., DONG, X., QU, Z., LU, S., HU, X., RUAN, S., LUO, S., WU, J.,

PENG, L., CHENG, F., PAN, L., ZOU, J., JIA, C., WANG, J., LIU, X., WANG, S., WU, X., GE, Q., HE, J., ZHAN, H., QIU, F., GUO, L., HUANG, C., JAKI, T., HAYDEN, F. G., HORBY, P. W., ZHANG, D. & WANG, C. 2020. A Trial of Lopinavir-Ritonavir in Adults Hospitalized with Severe Covid-19. *N Engl J Med*.

CHEN, N., ZHOU, M., DONG, X., QU, J., GONG, F., HAN, Y., QIU, Y., WANG, J., LIU, Y., WEI, Y., XIA, J., YU, T., ZHANG, X. & ZHANG, L. 2020. Epidemiological and clinical characteristics of 99 cases of 2019 novel coronavirus pneumonia in Wuhan, China: a descriptive study. *Lancet,* 395**,** 507-513.

CHEN, R. T., Y; ZHONG, X; LIANG, Y; LI, B; TAO, X; LIAO, B; 2020. Efficacy analysis of hyperbaric oxygen therapy in the treatment of severe coronavirus disease 2019 patients. *Acad. J. Second Mil. Med. Univ.,* 6**,** 604-611.

DONG, H., LI, J., LV, Y., ZHOU, Y., WANG, G., HU, S., HE, X., YANG, P., ZHOU, Z., XIANG, X. & WANG, C. Y. 2013. Comparative analysis of the alveolar macrophage proteome in ALI/ARDS patients between the exudative phase and recovery phase. *BMC Immunol,* 14**,** 25.

GENG, M., ZHOU, L., LIU, X. & LI, P. 2015. Hyperbaric oxygen treatment reduced the lung injury of type II decompression sickness. *Int J Clin Exp Pathol,* 8**,** 1797-803.

GILL, A. L. & BELL, C. N. 2004. Hyperbaric oxygen: its uses, mechanisms of action and outcomes. *QJM,* 97**,** 385-95.

GORENSTEIN, S. A., CASTELLANO, M. L., SLONE, E. S., GILLETTE, B., LIU, H., ALSAMARRAIE, C., JACOBSON, A. M., WALL, S. P., ADHIKARI, S., SWARTZ, J. L., MCMULLEN, J. J. S., OSORIO, M., KOZIATEK, C. A. & LEE, D. C. 2020. Hyperbaric oxygen therapy for COVID-19 patients with respiratory distress: treated cases versus propensity-matched controls. *Undersea Hyperb Med,* 47**,** 405-413.

GRASSELLI, G., PESENTI, A. & CECCONI, M. 2020. Critical Care Utilization for the COVID-19 Outbreak in Lombardy, Italy: Early Experience and Forecast During an Emergency Response. *JAMA*.

GUO, Y. R., CAO, Q. D., HONG, Z. S., TAN, Y. Y., CHEN, S. D., JIN, H. J., TAN, K. S., WANG, D. Y. & YAN, Y. 2020. The origin, transmission and clinical therapies on coronavirus disease 2019 (COVID-19) outbreak - an update on the status. *Mil Med Res,* 7**,** 11.

HAN, C. H., ZHANG, P. X., XU, W. G., LI, R. P., XU, J. J. & LIU, W. W. 2017. Polarization of macrophages in the blood after decompression in mice. *Med Gas Res,* 7**,** 236-240.

KARSHOVSKA, E., WEI, Y., SUBRAMANIAN, P., MOHIBULLAH, R., GEISSLER, C., BAATSCH, I., POPAL, A., CORBALAN CAMPOS, J., EXNER, N. & SCHOBER, A. 2020. HIF-1alpha (Hypoxia-Inducible Factor-1alpha) Promotes Macrophage Necroptosis by Regulating miR-210 and miR-383. *Arterioscler Thromb Vasc Biol,* 40**,** 583-596.

KJELLBERG, A., DE MAIO, A. & LINDHOLM, P. 2020. Can hyperbaric oxygen safely serve as an anti-inflammatory treatment for COVID-19? *Medical Hypotheses,* 144.

LICHTENSTEIN, D. 2014. Lung ultrasound in the critically ill. *Curr Opin Crit Care,* 20**,** 315-22.

MACA, J., JOR, O., HOLUB, M., SKLIENKA, P., BURSA, F., BURDA, M., JANOUT, V. & SEVCIK, P. 2017. Past and Present ARDS Mortality Rates: A Systematic Review. *Respir Care,* 62**,** 113-122.

MOON, R. E. (ed.) 2019. *Hyperbaric Oxygen Therapy Indications*: Undersea and Hyperbaric Medical Society.

OYAIZU, T., ENOMOTO, M., YAMAMOTO, N., TSUJI, K., HORIE, M., MUNETA, T., SEKIYA, I., OKAWA, A. & YAGISHITA, K. 2018. Hyperbaric oxygen reduces inflammation, oxygenates injured muscle, and regenerates skeletal muscle via macrophage and satellite cell activation. *Sci Rep,* 8**,** 1288.

PAGANINI, M., BOSCO, G., PEROZZO, F. A. G., KOHLSCHEEN, E., SONDA, R., BASSETTO, F., GARETTO, G., CAMPORESI, E. M. & THOM, S. R. 2020. The Role of Hyperbaric Oxygen Treatment for COVID-19: A Review. *Adv Exp Med Biol*.

PAN, F., YE, T., SUN, P., GUI, S., LIANG, B., LI, L., ZHENG, D., WANG, J., HESKETH, R. L., YANG, L. & ZHENG, C. 2020. Time Course of Lung Changes On Chest CT During Recovery From 2019 Novel Coronavirus (COVID-19) Pneumonia. *Radiology***,** 200370.

SULKOWSKI, S., SULKOWSKA, M., GIEDROJC, J., TERLIKOWSKI, S., DZIECIOL, J. & FAMULSKI, W. 1997. Evaluation of the effect of macrophage system activation on the intensity degree of early destructive changes in acute enzymatic lung injury. *Rocz Akad Med Bialymst,* 42 Suppl 1**,** 412-21.

THIBODEAUX, K., SPEYRER, M., RAZA, A., YAAKOV, R. & SERENA, T. E. 2020. Hyperbaric oxygen therapy in preventing mechanical ventilation in COVID-19 patients: a retrospective case series. *J Wound Care,* 29**,** S4-S8.

THOM, S. R. 2011. Hyperbaric oxygen: its mechanisms and efficacy. *Plast Reconstr Surg,* 127 Suppl 1**,** 131S-141S.

WENG, Y. S., TSENG, H. Y., CHEN, Y. A., SHEN, P. C., AL HAQ, A. T., CHEN, L. M., TUNG, Y. C. & HSU, H. L. 2019. MCT-1/miR-34a/IL-6/IL-6R signaling axis promotes EMT progression, cancer stemness and M2 macrophage polarization in triple-negative breast cancer. *Mol Cancer,* 18**,** 42.

YANG, J., ZHENG, Y., GOU, X., PU, K., CHEN, Z., GUO, Q., JI, R., WANG, H., WANG, Y. & ZHOU, Y. 2020a. Prevalence of comorbidities in the novel Wuhan coronavirus (COVID-19) infection: a systematic review and meta-analysis. *Int J Infect Dis*.

YANG, Y., PENG, F., WANG, R., GUAN, K., JIANG, T., XU, G., SUN, J. & CHANG, C. 2020b. The deadly coronaviruses: The 2003 SARS pandemic and the 2020 novel coronavirus epidemic in China. *J Autoimmun***,** 102434.

ZHANG, H., ZHOU, P., WEI, Y., YUE, H., WANG, Y., HU, M., ZHANG, S., CAO, T., YANG, C., LI, M., GUO, G., CHEN, X., CHEN, Y., LEI, M., LIU, H., ZHAO, J., PENG, P., WANG, C. Y. & DU, R. 2020. Histopathologic Changes and SARS-CoV-2 Immunostaining in the Lung of a Patient With COVID-19. *Ann Intern Med*.

ZHONG, X. T., X; TANG, Y; CHEN, R; 2020. The effect of hyperbaric oxygen therapy on hypoxia in patients with severe new coronavirus pneumonia: the first report. *Chinese Journal of Nautical Medicine and Hyperbaric Medicine*.

ZHOU, F., YU, T., DU, R., FAN, G., LIU, Y., LIU, Z., XIANG, J., WANG, Y., SONG, B., GU, X., GUAN, L., WEI, Y., LI, H., WU, X., XU, J., TU, S., ZHANG, Y., CHEN, H. & CAO, B. 2020. Clinical course and risk factors for mortality of adult inpatients with COVID-19 in Wuhan, China: a retrospective cohort study. *Lancet,* 395**,** 1054-1062.

Study Code: COVID-19-HBO
Version No: v.4
Date: 2021-02-27
EudraCT No: 2020-001349-37

17. Amendments and Administrative changes

The following amendments and Administrative changes have been made to this protocol since day of preparation:

Amendment	Section/Page	Date	Type/comment
Version 1		2020-04-23	Approved by IEC/IRB Sweden
Version 2<br>Spelling/layout errors, change of biobank, German sites added, change in screening procedure	 2.2, 5.3.3, 8.2, 9.1.1, 14.1.9, 14	2020-05-17	Non-substantial revision Sweden, Changed before inclusion of first subject.<br>IEC/IRB Germany submission.
Version 3<br>ClinicalTrials.gov identification<br>New sites Karolinska and Sahlgrenska<br>Change of NCI in Germany<br>New site Brasov<br>Added names of DSMB<br>Change of biobank<br>Revision history added<br>Updated background with new publications, updated references<br>Change of minor spelling/grammatical and layout errors and minor clarifications/explainantions in procedures	 Front page<br>Contact info/7-9<br><br><br><br>5.3.3<br>17<br>2.2, 16<br><br>Full protocol, update list of content	2020-11-16	Substantial revision/<br>IEC amendment Sweden, Germany<br>First submission IEC/IRB Romania
Version 4<br>Removed sites UCSD and Brasov, change of NCI Germany<br>Change in risk evaluation<br>Change in procedures:<br>-NEWS not mandatory x3<br>-ABG/CBG if warranted<br>-Increased limits for NEWS and AGB/CBG<br>Update responsible of Biobank<br>Clearification of withdrawal criteria: negative SARS-CoV-2<br>Recommended dose profile<br>Change of timeframe for AE reporting<br>Update of safety analysis to comply with DSMB instruction 10.6<br>Clearification of source data<br>Clearification of ICF process<br>Change of minor spelling/grammatical and layout errors and minor clarifications/explainations in procedures	 Contact info/7-8<br><br>3.5<br>5.2.1, 5.2.2<br><br><br><br>5.3<br>6.4<br><br>7.2<br>8.3.1<br><br>9.2.6.2<br><br><br>10.3<br>11.3<br>Full protocol, update list of content and revision history	2021-02-27	Substantial revision/<br>IEC amendment Sweden<br>IEC/IRB Germany re-submission.

50 (50)
